# Optical observation of needles in upward lightning flashes

**DOI:** 10.1038/s41598-020-74597-6

**Published:** 2020-10-15

**Authors:** Marcelo M. F. Saba, Amanda R. de Paiva, Luke C. Concollato, Tom A. Warner, Carina Schumann

**Affiliations:** 1 INPE–National Institute for Space Research, Av. dos Astronautas, S. José dos Campos, 1758 Brazil; 2 Department of Physics, University of Oxford, Oxford, UK; 3 ZT Research, Rapid City, SD USA; 4 Johannesburg Lightning Research Laboratory, School of Electrical and Information Engineering, University of the Witwatersrand, Johannesburg, South Africa

**Keywords:** Natural hazards, Plasma physics, Atmospheric dynamics, Imaging techniques

## Abstract

Why lightning sometimes has multiple discharges to ground is an unanswered question. Recently, the observation of small plasma structures on positive leaders re-ignited the search. These small plasma structures were observed as pulsing radio sources along the positive leader length and were named “needles”. Needles may be the missing link in explaining why lightning flickers with multiple discharges, but this requires further confirmation. In this work we present the first optical observations of these intriguing plasma structures. Our high-speed videos show needles blinking in slow motion in a sequential mode. We show that they are formed at unsuccessful leader branches, are as bright as the lightning leaders, and report several other optical characteristics.

## Introduction

The needle-like structures analyzed by Hare et al.^1^, Pu and Cummer^2^ and Shao et al.^3^, revealed some new details of the positive leader propagation to explain why lightning sometimes has multiple discharges. Needles could cause enough instability on the lightning channel current (see also Williams and Montanyá^4^) leading to a current cut-off that could enable the presence of recoil leaders and thus the formation of further strokes in the lightning flash (more information on current cut-off and recoil leaders in Heckman^5^, Williams^6^, Williams and Heckman^7^, Saba et al.^8^, Mazur et al.^9^ Warner et al.^10^, Tran and Rakov^11^). Their analysis was based on radio frequency signal observations of incloud propagation of positive leaders from intracloud or negative cloud-to-ground flashes. In this work, needles were observed by high-speed cameras during the upward propagation of positive leaders in three upward flashes.

During summer seasons between 2008 and 2018, monochrome high-speed cameras were pointed toward several towers located in Rapid City, SD, USA, and Sao Paulo, SP, Brazil. Rapid City is located in the northern High Plains of the United States and São Paulo city in the southeastern region of Brazil.

Six different high-speed digital video cameras (Photron Fastcam 512 PCI, Phantom v7.1, v7.3, v310, v711, and Miro 4) have been used to record images of upward flashes. All upward flashes were triggered by another discharge, most of them positive cloud-to-ground flashes.

All video imagery was time stamped to GPS with time resolutions and exposure times ranging from 10 μs (100,000 images per second) to 1 ms (1000 images per second). The minimum recording length of all the cameras was 1.6 s.

More than 170 upward flashes were recorded and analyzed (70% in Brazil and 30% in USA). Although the upward flashes and observing conditions were very similar in Brazil and in USA, needles were observed in only three upward flashes recorded in USA.

A detailed description of the cameras, the location of the towers and further details for the upward flashes are given in Warner^12^, Warner et al.^13^, Warner et al.^14^, Saba et al.^15^ and in the Supplementary Table S1.

An upward flash starts when the intensification of the electric field over a tall structure initiates an upward propagating leader from the tip of the structure. As reported by past studies using high-speed cameras and electric-field antennas in the USA and in Brazil, the initiation of positive leaders is usually triggered by a nearby flash activity^13,15,16^. Once the upward leader starts its propagation toward the cloud base, it may or may not branch before reaching the cloud base. Eventually, the channel luminosity decays and the upward flash is over. In some cases (approximately 25%), one or more sequences of dart-leader-return-stroke may occur after a period of no-luminosity along the channel^14,15^. The dart-leader is formed by the fast-negative leader from a bidirectional and bipolar discharge that develops in a small region along the path of the decayed positive leader that was in the cloud. This bidirectional and bipolar leader discharge is called a recoil leader and appears to play a key role in the existence of subsequent strokes in a negative cloud-to-ground flash (Saba et al.^8^, Mazur et al.^9^, Warner et al.^10^, Tran and Rakov^11^).

**Figure 1:**
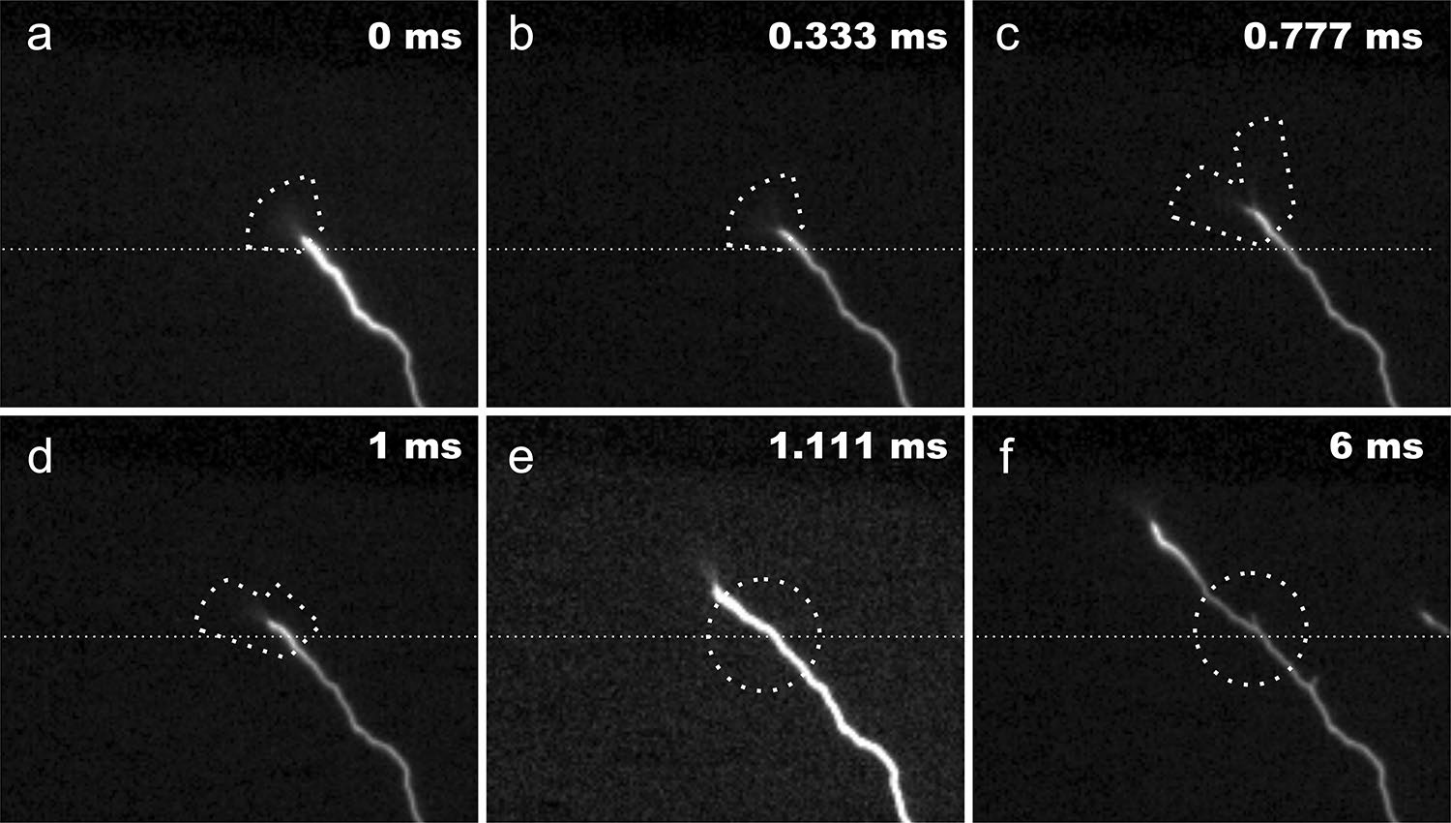
Formation and evolution of a needle as seen by high-speed cameras (Case 1—image exposure time of 111 µs). (**a**) Corona brush can be seen as positive leader propagates upwards. (**b**) Leader continues to propagate in the direction of corona brush. (**c**) The corona brush splits. (**d**) The leader propagates in one direction leaving a pocket of ionized air behind. (**e**,**f**) As the leader propagates further, a needle appears exactly at the portion of ionized air left behind when the corona brush split.

**Figure 2:**
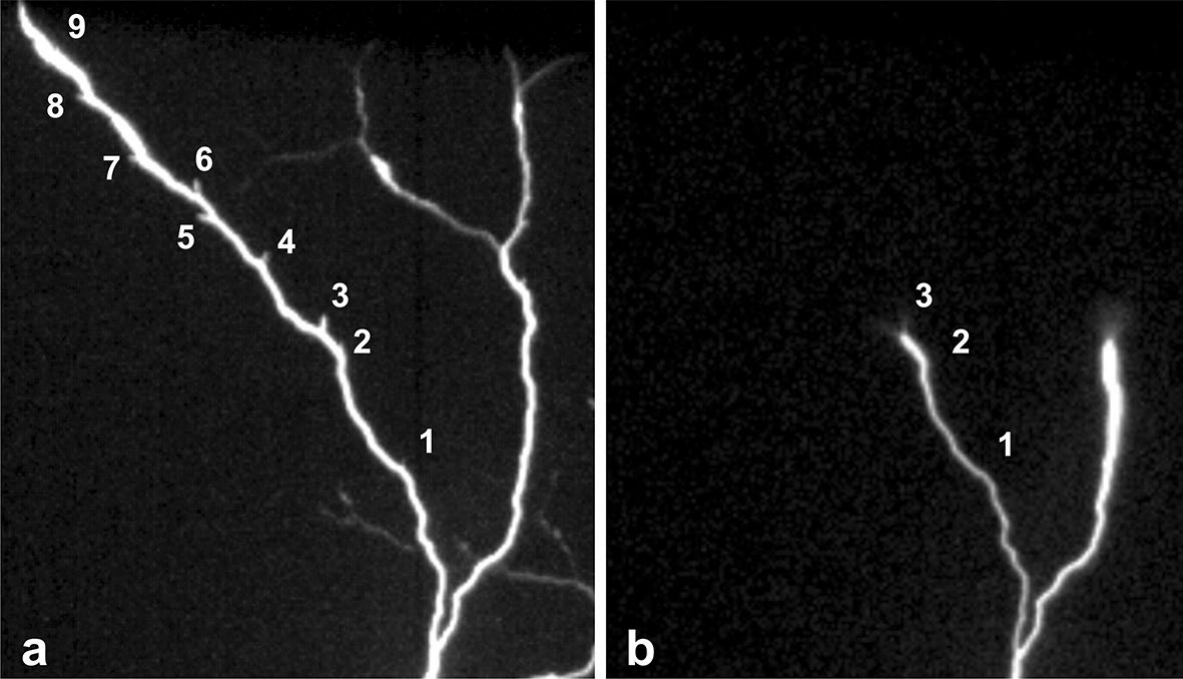
Needles along a positive branch, (**a**) Nine needles in a time integrated image of an upward flash. A tenth needle was also observed but not shown here because it was too dim. (**b**) The upward leader right before the formation of needle number 3. It shows a normal corona brush (on the right branch), and the split corona brush (on the left branch) that generated needle number 3 on the left image (from Case 1—exposure time of 111 µs).

When a positive leader is close to a sensitive high-speed video camera, it is sometimes possible to see a low-luminosity conic brush at the tip of the leader^17^. It is called corona brush and is believed to be formed by the electron convergence and corona streamer formation due to an intense electric field at the head of the positive leader. High-speed videos of upward and downward lightning recorded in the USA and in Brazil show that corona brushes are most seen at the tip of non-branched upward and downward positive leaders. During the tortuous path of the leader, they change direction and angle width and may also split prior to an unsuccessful (or successful) branch attempt (Figs. 1 and 2).

In the case of an unsuccessful branch after a brush split, the high-speed videos reveal that the splitting of the corona brush may give rise to bright and very short length leader segments. They form at an upward angle extending outward from the leader channel. They are as bright as the positive upward leader and much brighter than the corona brush (Figs. 1 and 2). These leader segments first appear some milliseconds after the corona brush splitting and they pulsate as the leader propagates upward (see Supplementary Video). These structures are identified as needles due to the high degree of similarity with the ones observed with VHF detection by Hare et al.^1^, Pu and Cummer^2^ and Shao et al.^3^.

As many as 11 pulsating segments as bright as the positive leader tip were seen along the leader channel as it propagated. With one exception, all pulses lasted less than 100 µs. The lengthiest needle, 73 m long (Case 2 in Supplementary Table S2), lasted 3 frames. The average 2D speed of this needle (2.7 × 10^5^ m s^−1^) is very similar to what was reported by Hare et al.^1^, Pu and Cummer^2^ (3 × 10^5^ m s^−1^ and 1–10 × 10^5^ m s^−1^ respectively) and also exhibit clear outward propagation from the positive leader channel. The average 2D length of these structures is 14 m and the distance between them 80 m (Supplementary Table S2). They pulse without growing in length. However, on one occasion, a short-lived negative leader branch developed from one of these “needles” after multiple preceding pulses.

**Figure 3:**
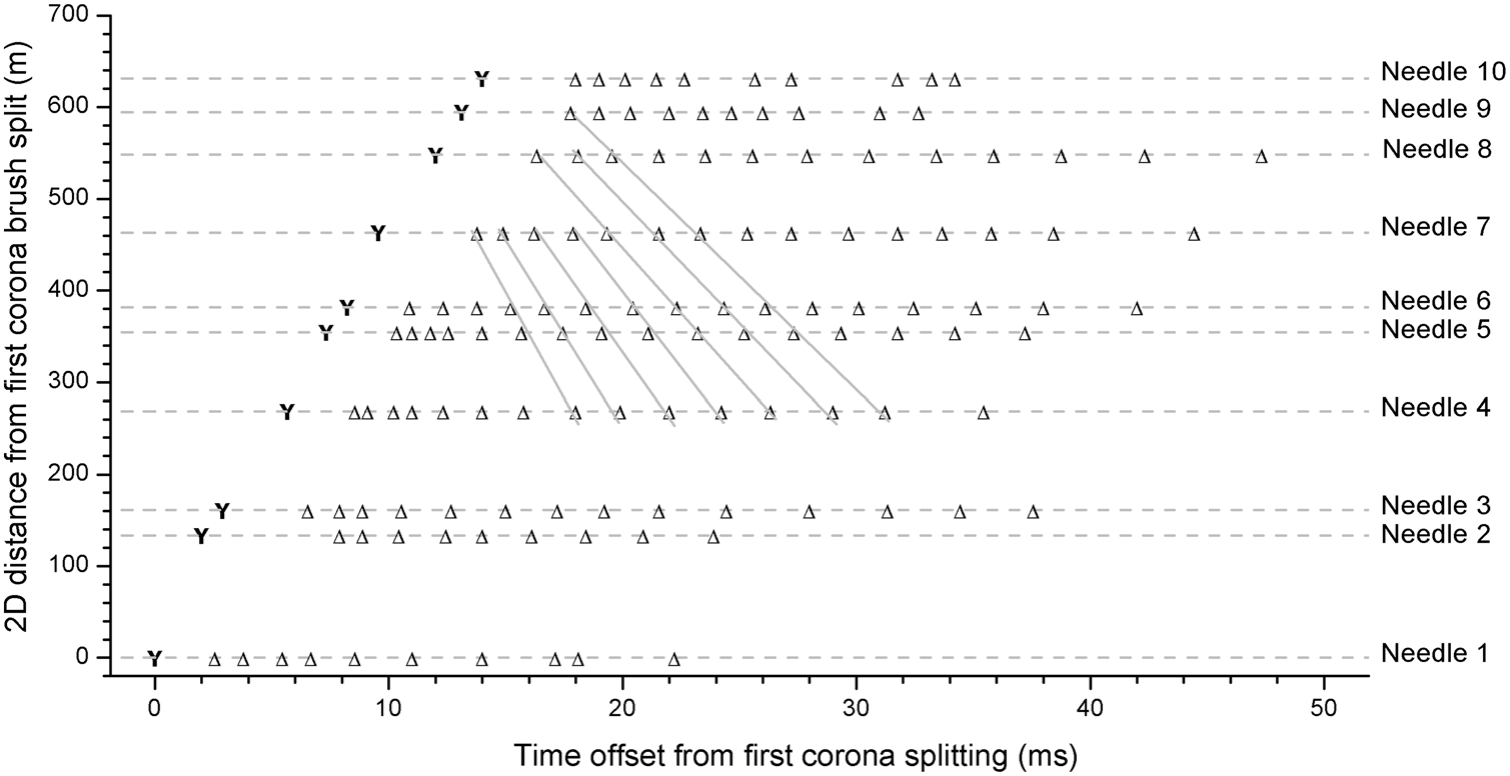
Occurrence of needles during the upward leader propagation. The letter “Y” in the beginning of each line corresponds to time when the corona brush split (and the branching failed). Each subsequent triangle on the line indicates pulses of the corresponding needle. The gray angled lines show the apparent temporal sequencing that may be occurring.

Figure 3 shows the time occurrence of each of 10 needles during the upward positive leader propagation shown in Fig. 2. Each line corresponds to one of the needles observed and numbered in Fig. 2. Note that they appear in a sequential way as the positive leader propagates upward at an average 2D speed of 4.2 × 10^4^ m s^−1^ (see Supplementary Video). Some needles pulsed only 9 times whereas others pulsed up to 16 times. Note also that the average time interval between the brush splitting and the first needle pulse (4.1 ms) is larger than the average pulsating period of the needle (2.1 ms). See Supplementary Table S2 and Supplementary Fig. S1 for more details.

A possible explanation as to why needles do not immediately appear after an unsuccessful leader branching, but rather only after the leader has propagated an average time of 4 ms, is that certain conditions are required for the needle to occur. A possible condition could be a minimum potential difference between the tip of the leader and the location of a needle that is achieved only after further propagation of the leader. The observed distance from the upward positive leader tip to the location of the first needle can be of hundreds of meters (more details shown in the Supplementary Table S1).

Although the pulse interval of a needle is shorter than the time interval between the brush corona split and the first needle appearance, the pulse interval of a needle increases with time (Supplementary Fig. S2) as also observed by Pu and Cummer^2^. It is unclear why this behavior occurs, but it may be tied to the lengthening of the leader and the increasing number of needles. If a minimum potential difference is required between the leader tip and each needle for a pulse to occur, this process may take an increasingly longer time to achieve with the increasing distance between them.

Needles also appear to have their later pulses following a temporal sequence that travels down the channel away from the leader tip (see Supplementary Video). This behavior is highlighted with the angled gray lines in Fig. 3. The speed of this apparent sequential illumination of the needles is approximately 2–5 × 10^4^ m s^−1^, similar to the upward speed of the positive leader. However, this apparent sequential illumination may be an observational artifact as it is not observed during the whole process. More observations are needed.

Needles are not a commonly observed feature. Out of more than 170 inspected upward flashes, needles were observed only in 3 events. It could be the case that needles are always present but not always observed. However, considering that observations were made in the USA and in Brazil in approximately the same conditions (similar high-speed cameras and distance from the upward flashes) we do not think that this is a reasonable hypothesis. If this were true, we would expect to see a broad range of needle luminosity, some dim and some bright ones. However, needles were almost always bright.

Commenting on the Hare et al.^1^ paper, Williams and Monatanyá^4^ states that it would be valuable to establish the connection between the formation of needles and the development of recoil leaders in the positive leader. High speed camera, as reported here, can see the positive leader, the recoil leaders and needles. However, recoil leaders were never observed in association with needle appearance. In all three cases, recoil leaders did not occur along the branch where the needles took place. In the case illustrated in Figs. 1 and 2, one recoil leader happened but only 121 ms after the last needle pulse. This implies that they are not associated with the third electric field reversal hypothesis made by Hare et al.^1^, in their Supplementary Information (according to this hypothesis when recoil leaders propagates by a needle, they could deposit enough negative charge to initiate a breakdown causing a needle twinkle). Also, no optical evidence of any disconnection was found on the positive leader.

The average size and flickering time interval of the needles analyzed here are shorter than what has been reported in previous works^1,2^. The average period and size were 2.6 ms and 14.3 m respectively (more information in Supplementary Table S2), whereas in Hare et al.^1^, sizes ranged from 30 to 100 m and the period from 3 to 7 ms, and in Pu and Cummer^2^ the reported size was 60 m and the period ranged from 6 to 7 ms. This could be due to the different ambient electric field where the needles occurred. In the work by Hare et al.^1^ and Pu and Cummer^2^, they all happened during the incloud propagation of positive leaders at a height of 5–7 km. In this work, they were observed during the upward propagation of positive leaders in upward flashes at a height between 0.3 and 1.9 km.

Therefore, the fact that needles are not common nor associated with the initiation or presence of recoil leaders suggests that they are not a key element that contributes to the presence of subsequent stroke in negative cloud-to-ground flashes. However, given that one needle appeared to transition into a self-propagating negative leader suggests that they could lead to opposite polarity leader branching.

**Figure 4:**
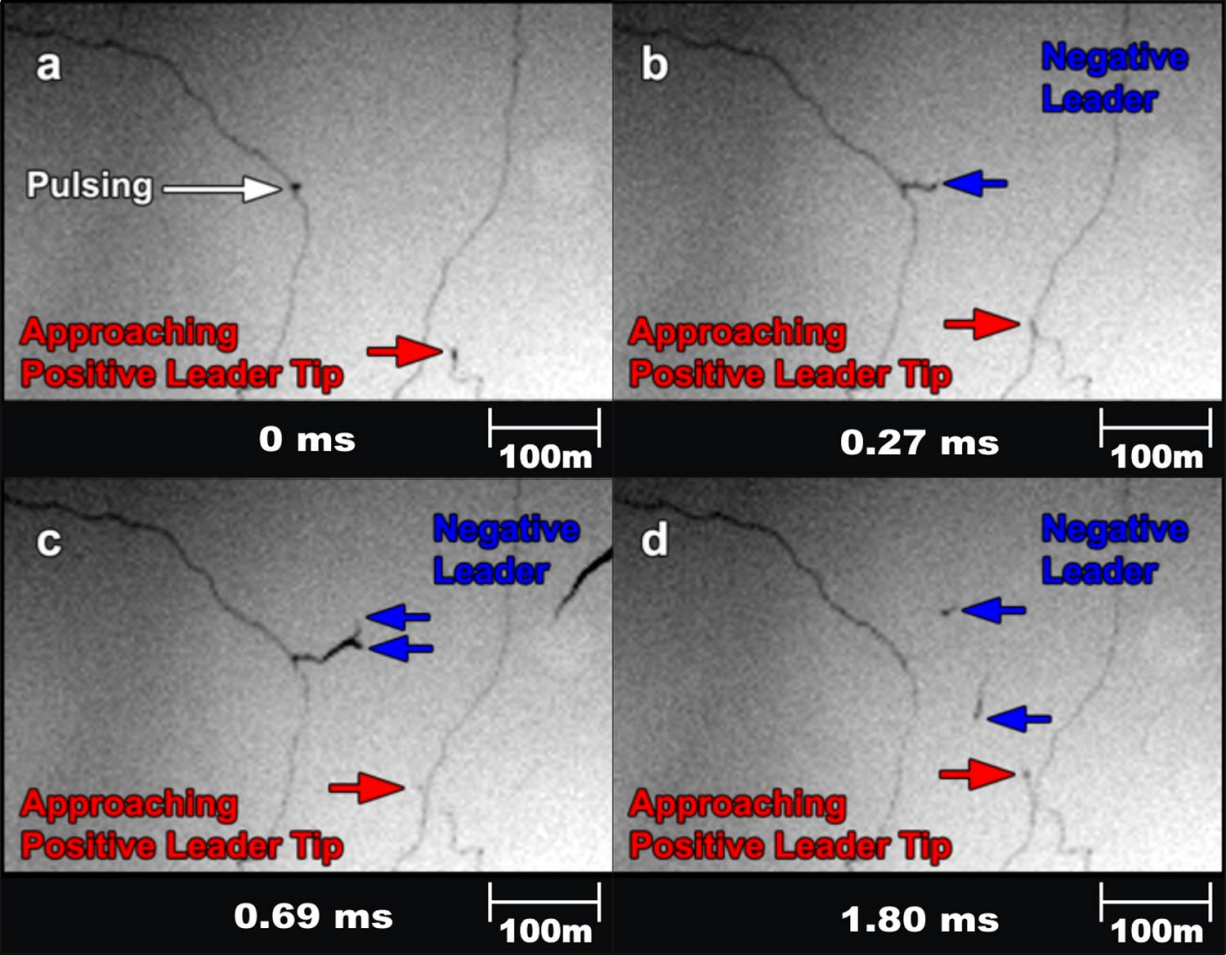
High-speed camera images (exposure time of 139 µs) of one needle transitioning into a self-propagating negative leader. (**a**) Pulsing of a needle and the approach of an upward propagating positive leader branch. (**b**) A negative leader initiates from the needle location as the positive leader tip continues to approach. (**c**,**d**) The negative leader branches and continues to propagate as the positive leader approaches further.

We have observed one case in which a negative leader developed from the location of a pulsing needle. During an upward flash on 29 May 2008, one of the positive leader branches developed a needle that pulsed 11 times. 29.0 ms after the branch attempt and 20.5 ms after the first of 11 needle pulses occurred at the location indicated in Fig. 4a, a negative leader initiated from the needle location. The initiation coincided with the approach of an upward propagating positive leader branch as indicated in Fig. 4b. The negative leader exhibited an average speed of 1 × 10^5^ m s^−1^, an erratic directional change and stepping which are defining behaviors for negative leaders when observed by high-speed cameras^10^ (see video of Case 2 in Supplementary Information). This negative leader branched once before decaying 4.0 ms after its initiation (Fig. 4c,d). This is the only optical observation we have obtained of a negative leader initiation from a pulsing needle site but serves to confirm the observations of negative leader initiation from needle sites as reported by Pu and Cummer^2^.

Clearly, more observations and research into this phenomenon are needed. We believe that the first optical observations of needles presented in this work add valuable information on these recently discovered plasma structures. It shed some light on when and where needles are formed along the positive leader, how they flicker and how frequent they are. Also, their relationship with positive leader propagation and the presence of recoil leaders may help to understand why negative cloud-to-ground lightning have multiple strokes.

## Supplementary information

Supplementary Information 1.

Supplementary Information 2.

## Data Availability

The original raw videos are available at https://urlib.net/rep/8JMKD3MGPGW/42CANS2.
