# Supplementary material for: Optical observation of needles in upward lightning flashes
Source: Sci Rep. 2020 Oct 15;10:17460. doi: 10.1038/s41598-020-74597-6 (PMC7562907; doi:10.1038/s41598-020-74597-6)
Supplement: Supplementary file 1 — Supplementary Information 1. [file 41598_2020_74597_MOESM1_ESM.pdf]

Optical observation of needles in upward lightning flashes

Marcelo M. F. Saba, Amanda R. de Paiva, Luke C. Concollato, Tom A. Warner, Carina Schumann

Supplementary Videos

The following videos are available at http://urlib.net/rep/8JMKD3MGPGW/42CANS2:

1. Video of one upward lightning containing needles showing: a) a positive leader propagating upward; b) a close-up showing the splitting of the corona brush at the tip of positive leader and the needle formation; c) needles flickering in a sequential way.
2. Videos of Case 1, Case 2, and Case 3 in their original format.

Supplementary Table S1. Presents some characteristics of the cameras (location and settings) and of the needles observed in three upward flashes during the upward propagation of the positive leader. One of the upward flashes (Case 3) had simultaneous upward leaders from two close towers and was divided into Case 3a and Case 3b.

General characteristics	Case 1	Case 2	Case 3a	Case 3b
Date and time (UT) of upward flash	24 May 2010<br>07:31:50	29 May 2008<br>22:38:51	30 Aug 2011<br>04:19:24	
Distance from camera (km)	4.6	4.8	0.88	0.63
High-speed camera (spectral response from 370-1100 nm)	Phantom v310	Phantom v7.1	Phantom v310	
Lens used	Sigma 20 mm, f/1.8	Nikon 28 mm, f/1.4	Fujinon 6.5 mm, f/2	
Resolution of the image (meters/pixel)	4.63	3.76	1.80	2.51
High speed camera frame rate (images per second)	9,000	7,207	10,000	
Frame interval (µs)	111.11	139.00	100.00	
Exposure time (µs)	110.49	135.00	99.32	
Number of needles	11	10	4	4
Time interval from leader initiation to first needle appearance (ms)	11.7	31.1	7.0	17.7
Distance from the tip of the positive leader to the first needle (m)	205	435	80	64

Height of first needle (m)	1056	900	260	582
Height of last needle (m)	1752	1196	564	726
2D distance between the first and last needle (m)	802	673	307	133
2D average speed of leader during the occurrence of needles ($x10^4$ $m.s^{-1}$)	4.2	8.7	3.0	1.9
Presence of recoil leaders during the occurrence of needles	121 ms after the last needle	Absent	Absent	Absent

Supplementary Table S2. Contains some statistical parameters of the needles for each of the upward flashes. It also presents the statistics of all needles from the upward flashes analyzed together. The correspondent histogram distribution for all cases is presented in Supplementary Figure S1.

	**Brush split to first needle pulse time interval (ms)**				**Flickering period (ms)**				**2D needle length (m)**				**2D distance between needles (m)**			
**Case**	**N**	**Min**	**Max**	**AM**	**N**	**Min**	**Max**	**AM**	**N**	**Min**	**Max**	**AM**	**N**	**Min**	**Max**	**AM**
**1**	11	2.5	7.4	4.1	126	0.6	6.0	2.1	11	4.6	23.2	13.8	10	18	182	80
**2**	10	3.0	25.0	11.2	63	0.7	33.7	3.7	8	3.8	73.0	17.7	9	20	128	75
**3a**	4	0.9	3.5	2.0	13	0.3	2.9	1.1	4	2.5	10.2	5.9	3	20	189	58
**3b**	4	2.8	5.3	3.9	30	0.8	9.8	2.9	4	2.5	28.1	16.9	3	16	110	52
**All**	**29**	**0.9**	**25.0**	**6.1**	**232**	**0.3**	**33.7**	**2.6**	**27**	**2.3**	**73.0**	**14.3**	**25**	**16**	**189**	**78**

Min stands for minimum, Max for maximum and AM for arithmetic mean value.

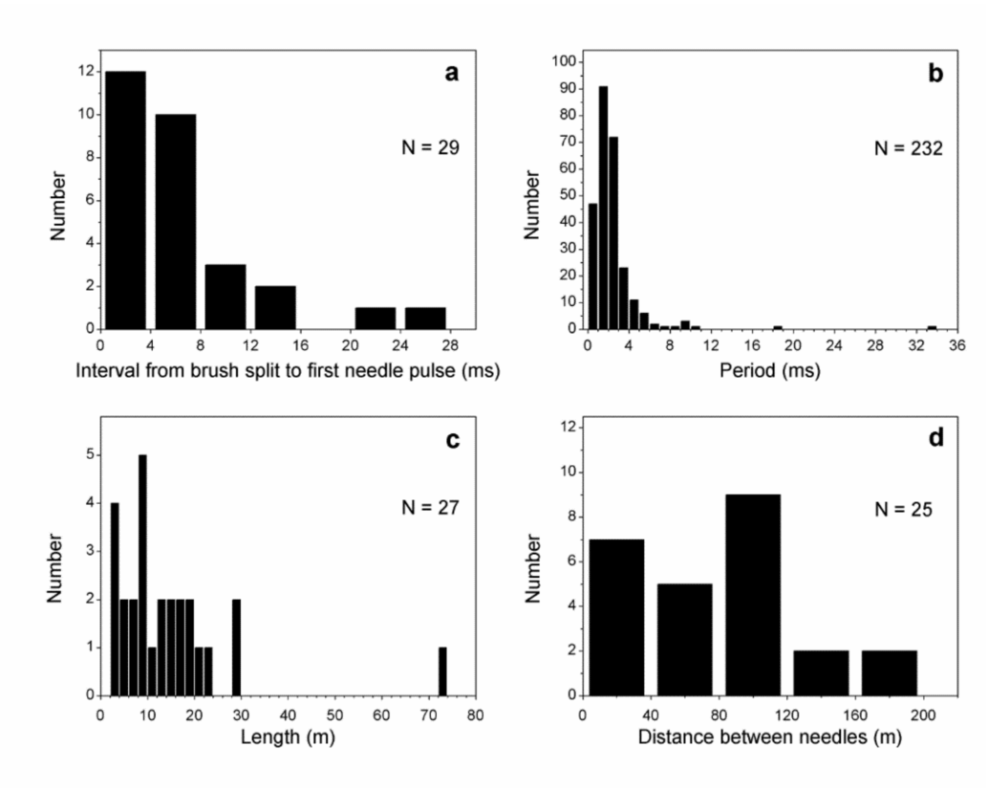

Supplementary Figure S1. Histogram of the needle parameters for all cases together. a, Time interval between corona brush split to first needle pulse. b, flickering period. c, needle length. d, distance between needles. N indicate the sample size used.

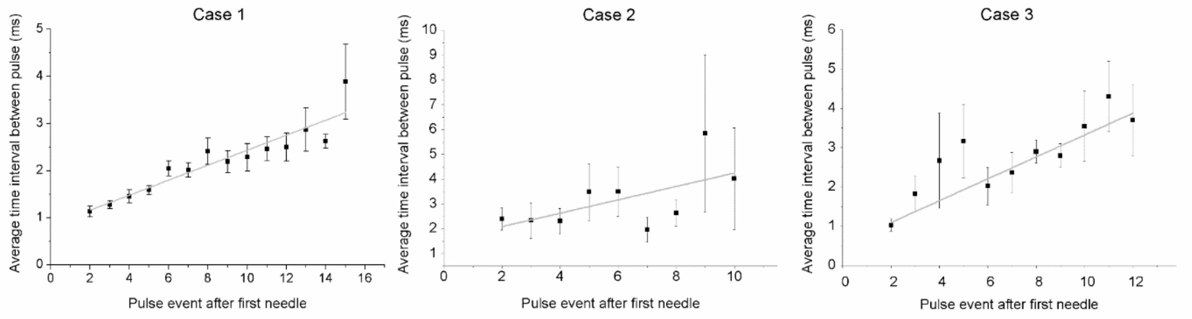

Supplementary Figure S2. Shows the average time interval between successive pulses of each needle of each flash. Interval number 1 corresponds to the time interval between the first and second pulse, number 2 to the time interval between the second and the third and so on. The gray line indicates the linear fit and the vertical bars represent the errors (standard deviation/sqrt [N]).
